# New Cytotoxic Azaphilones from *Monascus purpureus*-Fermented Rice (Red Yeast Rice)

**DOI:** 10.3390/molecules15031958

**Published:** 2010-03-18

**Authors:** Jin-Jie Li, Xiao-Ya Shang, Ling-Ling Li, Ming-Tao Liu, Jian-Quan Zheng, Zong-Lian Jin

**Affiliations:** 1 Beijing Key Laboratory of Bioactive Substances and Functional Foods, Beijing Union University, Beijing 100083, China; 2 Institute of Materia Medica, Chinese Academy of Medical Sciences and Peking Union Medical College, Beijing 100050, China

**Keywords:** *Monascus purpureus*, cytotoxicity, azaphilones, absolute configurations

## Abstract

Using a cell-based cytotoxicity assay three new cytotoxic azaphilones, including two stereoisomers and designated monapurones A-C (**1-3**), were isolated from the extract of *Monascus purpureus-*fermented rice (red yeast rice). Their structures were elucidated by detailed interpretation of spectroscopic and chemical data. The relative configurations were assigned on the basis of analysis of NOE data, and the absolute configurations were determined by direct comparison of their CD spectra with those of known azaphilones and chemical correlations. In the *in* v*itro* assays, monapurones A-C (**1-3**) showed selective cytotoxicity against human cancer cell line A549 with IC_50_ values of 3.8, 2.8 and 2.4 *μ*M respectively, while exhibiting no significant toxicity to normal MRC-5 and WI-38 cells at the same concentration.

## 1. Introduction

The fungus *Monascus* has traditionally been used to prepare red fermented rice, as a natural food colorant or food preservative of meat and used as a folk medicine in east Asia for centuries [1,2], Many reports indicated that metabolic products from fermentation of *Monascus* species were utilized to decrease blood pressure, lower plasma cholesterol levels and blood sugar. They also showed antitumor, antibacterial and antioxidative activities [3,4,5,6,7,8]. As part of a program to search for potential antitumor agents from natural sources, we investigated the extract of red yeast rice fermented with *Monascus purpureus*. From the petroleum ether-soluble portion of the EtOH extract of this material, three new cytotoxic azaphilones, including two stereoisomers, and designated monapurones A-C (**1-3**), were isolated. In the *in* *vitro* assays, monapurones A-C (**1-3**) showed selective cytotoxicity against human cancer cell line A549 with IC_50_ value of 3.8, 2.8 and 2.4 μM respectively, while exhibiting no significant toxicity to normal MRC-5 and WI-38 cells at the same concentration. To our knowledge, this is the first isolation of azaphilones with a C_20_ skeleton. Herein, we report the isolation, characterization and biological activity of monapurones A-C (**1-3**).

**Figure 1:**
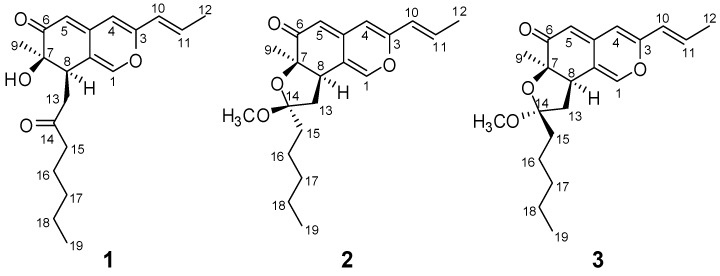
Chemical structures of compounds **1**, **2 **and **3**.

## 2. Results and Discussion

The petroleum ether portion with the highest cytotoxic activity ( IC_50_< 50 μg/mL ) was subjected to column chromatography over silica gel, Sephadex LH-20 and reverse-phase HPLC to yield three new azaphilones: monapurones A-C (**1-3**, Figure 1)

Monapurones A (**1**) was obtained as a yellow oil. Its IR absorptions at 3,403, 1,712, and 1,618 cm^‑1^ implied the existence of hydroxyl, carbonyl, and double bond functional groups, respectively. The UV spectrum showed absorption maxima at 354, 284 and 230 nm, indicating the presence of an extended conjugated system. High-resolution mass spectral analysis suggested a molecular formula C_20_H_26_O_4_ (*m*/*z* 330.1827 [M]^+^) with eight degrees of unsaturation, which was consistent with the structural information provided by ^1^H-NMR and ^13^C-NMR spectra. 

The ^1^H-NMR spectrum of **1 **displayed the typical pattern of an azaphilone skeleton [3,9] with three olefinic protons attributed to H-1, H-4, and H-5. In the lower field region, another two olefinic proton signals attributed to a disubstituted double bond at *δ* 5.94 (1H, dd, *J =* 1.5, 15.5 Hz, H-10) and 6.49 (1H, dq, *J =* 7.0, 15.5 Hz, H-11). In the higher field region, the ^1^H-NMR spectrum exhibited three methyl signals at *δ* 1.28 (3H, s, CH_3_-9), 1.89 (3H, dd, *J =* 1.0, 6.5 Hz, CH_3_-12) and 0.84 (3H, t, *J =* 7.5 Hz, CH_3_-19), and five methylene signals at *δ* [2.33 (1H, dd, *J =* 10.0, 18.0 Hz, H-13α), 2.99 (1H, dd, *J =* 3.0, 18.0 Hz, H-13*β*)], [2.22 (1H, dt, *J =* 7.5, 18.0 Hz, H-15α), 2.34 (1H, dt, *J =* 7.5, 18.0 Hz, H-15*β*)], 1.49 (2H, m, H_2_-16), 1.24 (2H, m, H_2_-17) and 1.18 (2H, m, H_2_-18) as well as one methine resonating at *δ* 3.37 (1H, dd, *J* =3.0, 10.0 Hz, H-8) (Table 1).

The ^13^C-NMR spectrum of **1** showed 20 carbons, and the DEPT experiment differentiated them to be 3 × CH_3_, 5 × CH_2_, 6 × CH, and 6 × C (Table 2). On the basis of the chemical shift values, 10 of them were assigned to be sp^2^ carbons including two carbonyls (*δ*_c_ 198.3, 209.8) and eight olefinic carbons, and 10 of them were sp^3^ carbons (one oxygen-bearing, *δ* > 70 ppm). The structure of **1 **was finally elucidated as azaphilone by analysis of the data obtained in 2D NMR experiments and comparison with those of previously characterized azaphilones [9,10,11,12].

**Figure 2:**
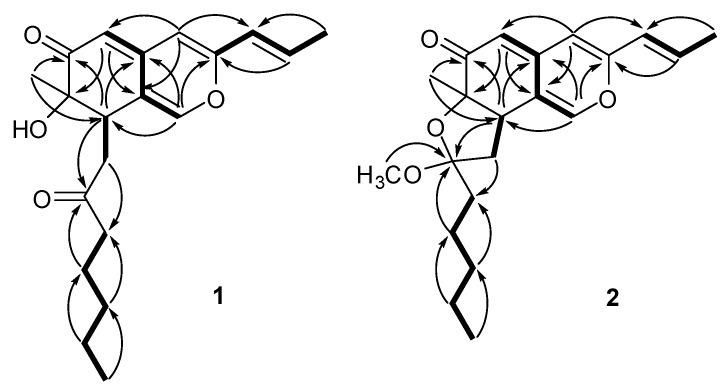
Main ^1^H-^1^H COSY (bold lines) and HMBC (arrows) correlations of compounds **1 **and **2**.

The proton and protonated carbon signals in the NMR spectra of **1 **were assigned unequivocally on the basis of heteronuclear correlations in the HMQC spectrum. In the HMBC spectrum of **1**, a series of two- and three-bond correlations from H-1 to C-3, C-8, C-4a, and C-8a, from H-4 to C-3, C-5, C-4a and C-8a, from H-5 to C-4, C-6, C-7, C-4a and C-8a, from CH_3_-9 to C-6, C-7 and C-8 (Figure 2, arrows), in combination with their chemical shift values, revealed unequivocally a 7-hydroxy-7-methyl-7,8-dihydro-6*H*- isochromen-6-one nucleus (azaphilone skeleton) for **1**. The presence of prop-1-enyl unit was established by ^1^H–^1^H coupling between H-11/H-10 and CH_3_-12 and long-range correlations between CH_3_-12 and C-10 and C-11. In addition, HMBC correlations from H-4 to C-10, from H-10 to C-3 and C-4 and from H-11 to C-3 allowed the prop-1-enyl unit to be connected to C-3 of the azaphilone nucleus. Futher investigation of ^1^H–^1^H COSY and HMBC spectra of **1** (Figure 2) revealed the presence of the 2-oxoheptyl side chain, which was located at C-8 of the azaphilone nucleus based on the homonuclear coupling correlation between H-8 and H_2_-13 and the long-range correlations from H-8 to C-13 and C-14, and from H_2_-13 to C-7, C-8 and C-8a. Thus, the full planar structure of **1 **was assigned as shown in Figure 1.

**Figure 3:**
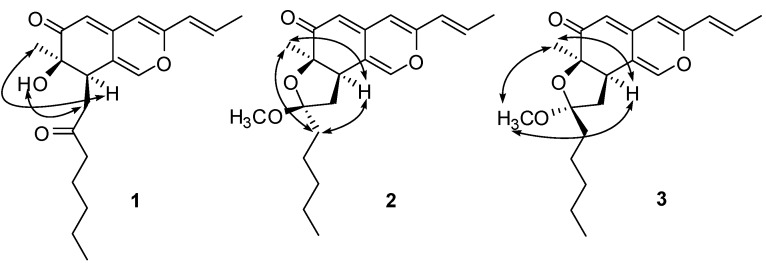
Key NOESY correlations of compounds **1**-**3**.

The relative stereochemistry of **1 **was verified by a careful analysis of its 600 MHz NOESY (in CD_3_COCD_3_) and 500 MHz NOESY (in CDCl_3_) spectroscopic data. NOE correlations between H-8 and CH_3_-9 revealed that these protons were cofacial and arbitrarily defined as having an α-orientation. The 2-oxoheptyl side chain and OH-7 were assigned to be *β*-configuration judging from the NOE correlations between OH-7 and H-13*β* (Figure 3). The C-10–C-11 double bond was established to be *E*-form by the coupling constant (Table 1) and the NOE correlation between H-10 and CH_3_-12. Furthermore, the absolute configuration of **1 **was determined by direct comparison of the CD spectrum, which showed the positive (369 nm and 258 nm) and negative (326 nm) Cotton effects, with those of known azaphilones. The positive Cotton effects at 369 nm (Δε +5.5) clearly showed (*R*)-configuration of chiral center at C-7 [9,12,13,14]. Since the chiral center at C-7 was *R*, C-8 was established as (*R*)-configuration by NOESY. Based on the above spectral evidence, monapurone A (**1**) was determined to be (7*R*,8*R*)-7-hydroxy-7-methyl-8-(2-oxoheptyl)-3-((*E*)-prop-1-enyl))-7,8-dihydro-6*H*-isochromen-6-one (Figure 1).

**Table 1:** ^1^H-NMR data forcompounds **1**-**3**.

No.	1^a^ *δ*_H _(mult, *J*, Hz)	1^b^ *δ*_H _(mult, *J*, Hz)	2^a^ *δ*_H _(mult, *J*, Hz)	3^a^ *δ*_H _(mult, *J*, Hz)
1	7.35 (br s)	7.37 (brs)	7.04 (br s)	7.09 (br s)
4	5.95 (br s)	6.23 (brs)	5.91 (br s)	5.90 (br s)
5	5.37 ( br s )	5.34 (d. 1.2)	5.37 (br s)	5.34 (br s)
8	3.37 (dd, 3.0, 10.0)	3.31 (dd, 3.6, 10.2)	2.92 (t, 10.0)	3.30 (t, 10.0)
9	1.28 (s)	1.21 (s)	1.31 (s)	1.36 (s)
10	5.94 (dd, 1.5, 15.5)	6.14 (dd, 1.8 15.6)	5.95 (dd, 1.5, 15.5)	5.93 (dd, 1.5, 15.5)
11	6.49 (dq, 7.0, 15.5)	6.50 (dq, 7.2, 15.6)	6.41 (dq, 7.0, 15.5)	6.43 (dq, 7.0, 15.5)
12	1.89 (dd, 1.0, 6.5)	1.88 (dd, 1.8, 6.6)	1.87 (dd, 1.0, 6.5)	1.86 (dd, 1.0, 6.5)
13α	2.33(dd, 10.0, 18.0)	2.39 (dd, 10.2, 17.4)	1.96 (dd, 10.5, 13.0)	1.93 (overlap)
13β	2.99 (dd, 3.0, 18.0)	2.99 (dd, 3.6, 17.4 )	2.36 (dd, 10.5, 13.0)	1.98 (overlap)
15a	2.22 (dt, 7.5, 18.0)	2.32 (dt, 7.5, 18.0)	1.58 (m)	1.45 (m)
15b	2.34 (dt, 7.5, 18.0)	2.39 (dt, 7.5, 18.0)	1.84 (m)	2.00 (overlap)
16	1.49 (m)	1.48 (m)	1.29 (m)	1.23 (m)
17	1.24 (m)	1.19 (m)	1.29 (m)	1.23 (m)
18	1.18 (m)	1.28 (m)	1.29 (m)	1.23 (m)
19	0.84 (t, 7.5)	0.84 (t, 7.8)	0.87 (t, 7.5)	0.82 (t, 7.5)
OH-7	4.17 (br s)	4.17 (br s)		
OCH_3_			3.16 (s)	3.29 (s)

^a ^Data were recorded in CDCl_3_ at 500 MHz; ^b ^Data were recorded in CD_3_COCD_3_ at 600 MHz. The assignments were based on DEPT, ^1^H-^1^H COSY, HMQC and HMBC experiments.

Monapurone B (**2**) was isolated as a yellow oil. Its molecular formula C_21_H_28_O_4_ was established by HREI-MS (*m*/*z* 344.1993 [M]^+^) corresponding to 8 degrees of unsaturation. This molecular formula was also supported by ^1^H- and ^13^C-NMR spectral data (Table 1 and Table 2).

The NMR spectral data of **2 **were similar to those of **1**, except that the carbonyl signal(*δ*_c_ 209.8, C-14) in **1** was replaced by a hemiacetal carbon signal (*δ*_c_ 108.0, C-14) in **2 **and C-7 of **2 **was downfield shifted by Δ*δ*_c_ 9.6 ppm compared to that of **1**. These data indicated the linkage between C-7 and C-14 via oxygen atom to form an five-membered ring in **2**, which was further confirmed by the 2D NMR experiments and the absence of hydroxyl absorption band at 3,403 cm^-1^ in the IR spectrum. Meanwhile, the NMR spectra of **2** showed the presence of an extra methoxyl signal (*δ*_H _3.16, *δ*_c_ 48.5), which was located at C-14 on the basis of the HMBC correlation from the methoxy protons at C-14 to C-14. 

The relative configuration of H-8, CH_3_-9 and the pentyl side chain were established to be on the same side of the molecular plane toward α-orientation based on their NOE correlations **(Figure 3**) between H-8 and CH_3_-9/CH_2_-15. As a consequence, the methoxyl group at C-14 (CH_3_O-14) was assigned as having a *β*-orientation. Furthermore, the absolute configuration at C-7 was determined to be *R* by comparing its CD spectrum with that of **1**, which showed both positive (357 and 259 nm) and negative (334 nm) Cotton effects. Therefore, monapurone B (**2**) was elucidated to be (6a*R*, 8*S*, 9a*R*)-8-methoxy- 6a-methyl-8-pentyl-3-((*E*)-prop-1-enyl))-6a, 8, 9, 9a-tetrahydro-6*H*-furo [2,3-*h*] isochromen-6-one (Figure 1).

**Table 2:** ^13^C-NMR data forcompounds **1**-**3**.

No.	1 *δ*_C, _mult	2 *δ*_C, _mult	3 *δ*_C, _mult	No.	1 *δ*_C, _mult	2 *δ*_C, _mult	3 *δ*_C, _mult
1	146.0 ( d )	143.4 ( d )	144.1 ( d )	11	134.3 ( d )	133.1 ( d )	133.6 ( d )
3	155.5 ( s )	154.7 ( s )	155.1 ( s )	12	18.4 ( q )	18.4 ( q )	18.4 ( q )
4	106.9 ( d )	108.2 ( d )	107.6 ( d )	13	40.4 ( t )	44.8 ( t )	43.3 ( t )
4a	145.8 ( s )	142.9 ( s )	143.6 ( s )	14	209.8 ( s )	108.0 ( s )	107.0 ( s )
5	103.7 ( d )	107.0 ( d )	106.7 ( d )	15	43.5 ( t )	34.9 ( t )	34.3 ( t )
6	198.3 ( s )	196.2 ( s )	196.8 ( s )	16	23.4 ( t )	24.1 ( t )	24.7 ( t )
7	73.1 ( s )	82.7 ( s )	83.6 ( s )	17	22.4 ( t )	24.0 ( t )	24.1 ( t )
8	40.5 ( d )	44.8 ( d )	43.5 ( d )	18	31.3 ( t )	31.9 ( t )	31.8 ( t )
8a	120.1 ( s )	118.1 ( s )	117.2 ( s )	19	13.9 ( q )	14.0 ( q )	13.9 ( q )
9	26.8 ( q )	22.5 ( q )	22.4 ( q )	OCH_3_		48.5	48.3
10	123.1 ( d )	123.3 ( d )	123.2 ( d )				

^13^C-NMR data were measured in CDCl_3_ at 125 MHz. The assignments were based on DEPT, ^1^H-^1^H COSY, HMQC and HMBC experiments.

Monapurone C (**3**) was obtained as a yellow oil. The ^13^C-NMR spectral data of **3 **was very similar to that of **2**, except that the chemical shifts of C-8, C-13 and C-14 in **3** were upfield shifted by ∆*δ*_c _1.3, 1.5 and 1.0 ppm, respectively, and C-7 of **3** was downfield shifted by ∆*δ*_c _0.9ppm, compared to those of **2**. The HREI-MS established its molecular formula as C_21_H_28_O_4 _identical to that of **2**. These information indicated it was isomeric with **2 **(Figure 1). In the NOESY spectrum of **3**, strong NOE correlations between H-8 and CH_3_-9, CH_3_O-14 clearly demonstrated that these protons were oriented on the same side of the tetrahydrofuran ring toward α-orientation, and confirmed the proposed structure as shown in Figure 3. The absolute configuration at C-7 was determined to be *R* by comparing the CD spectra of **1** and **2**. Combined with other 2D NMR experiments, monapurone C (**3**) was established as (6a*R*, 8*R*, 9a*R*)-8-methoxy-6a-methyl-8-pentyl-3-((*E*)-prop-1-enyl))-6a,8,9,9a-tetrahydro-6*H*-furo[2,3-*h*]- isochromen-6-one (Figure 1).

To verify the deduced stereochemistry at C-7 of **2** and **3**, the two compounds were subjected to acid hydrolysis with 0.1N HCl at room temperature. Almost all **2** and **3** were converted into **1 **after two hours. The products in both reactions were identified to be **1 **based on the identical ^1^H-NMR, ^1^H-^1^H COSY, NOESY and CD data. On the basis of the above results, the chiral centers at C-7 of **2** and **3** were further established as *R* configuration.

In the *in* *vitro* cytotoxic assays, monapurones A-C (**1-3**) showed potent selective cytotoxicity against human cancer cell line A549 with IC_50_ value of 3.8, 2.8 and 2.4 μM respectively, while posing no toxicity to normal MRC-5 and WI-38 cells at the same concentration. 

## 3. Experimental

### 3.1. General

CD spectra were recorded on a JASCO J-810 circular dichroism spectrometer. UV data were determined on a Hitachi UV-3000 spectrometer, and IR spectra were recorded as KBr disks on a Nicolet Impact 400 FT-IR Spectrophotometer. 1D- and 2D-NMR spectra were acquired in CDCl_3_ and CD_3_COCD_3_ with TMS as internal standard on Varian 500 or 600 MHz spectrometers. Mass spectra including high-resolution mass spectra were recorded on a JEOL JMS AX-500 spectrometer. Column chromatography was performed with silica gel (200-300 mesh, Qingdao Marine Chemical Inc. China), RP-18 reverse phase silica gel (43-60 μm) and Sephadex LH-20 (Pharmacia Biotech AB, Uppsala Sweden). LPLC separation was performed with Combiflash (ISCO Companion). HPLC was performed with a Waters 600 system with 2996 DAD-detector, with an Alltima (C18, 250 × 10 mm i.d., 5 μm) column. TLC was carried out with glass precoated silica gel GF254 plates. Spots were visualized under UV light or by spraying with 8 % H_2_SO_4_ in 95 % EtOH followed by heating.

### 3.2. Fungal material

Red yeast rice was prepared from cooked paddy rice inoculated with *Monascus purpureus* B0708, purchased from the Beijing Dawn Aerospace Bio-tech Company. The sample was deposited at Beijing Union University, Beijing Key Laboratory of Bioactive Substances and Functional Foods, Beijing 100083, China.

### 3.3. Extraction and isolation

The dried red yeast rice powder (4.5 kg) was extracted with 50 L of 95 % EtOH, 80 % EtOH and 60 % EtOH, respectively, at room temperature (×3, each for 3 hours). After removal of the solvent under reduced pressure, the residue (850.0 g) was suspended in 3 L of H_2_O, and then partitioned sequentially with petroleum ether (3 times with 5 L each) and EtOAc (4 times with 5 L each) to yield a petroleum ether (38.0 g), a EtOAc (223.5 g) and a H_2_O (588.5 g) fractions, respectively.

The petroleum ether-soluble portion (38.0 g) showing cytotoxic activity (IC_50_< 50 μg/mL) was fractionated via silica gel column chromatography eluted with a gradient of increasing acetone (0–100%) in petroleum ether (60–90 °C) to give 12 fractions F_1_-F_12_. The cytotoxic fraction F_3 _(6.5 g, IC_50_< 20 μg/mL) was further separated via Sephadex LH-20 eluted with petroleum ether-chloroform-acetone (5:5:1) to afford five subfractions. The first subfraction was purified by preparative reversed phase (C-18) HPLC eluted with ACN-H_2_O (40:60) to yield **1** (14.3 mg), **2** (12.6 mg) and **3** (11.8 mg). 

### 3.4. Acid hydrolysis of ***2*** and ***3***

Compound **2 **(5.0 mg in MeOH) was hydrolyzed with 0.1 N HCl (0.5 mL) at room temperature for 2 hours. The reaction progress was monitored by TLC eluted with CHCl_3_–CH_3_COCH_3_ (6:1). After evaporation of the solvent under a stream of nitrogen, the residue dissolved in CD_3_COCD_3_ with TMS as internal standard was analyzed by Varian 600 MHz spectrometer. Then, the solvent was removed under a nitrogen stream and the residue dissolved in MeOH was analyzed on the JASCO J-810 circular dichroism spectrometer. Compound **3 **(5.0 mg) was hydrolyzed using the same method.

### 3.5. Cells and culture condition

Human lung fibroblast (WI-38), human lung fibroblast (MRC-5), human colon cancer (HCT-8), human hepatoma (Bel7402), human stomach cancer (BGC-823), human lung adenocarcinoma (A549), human breast cancer (MCF-7), and human ovarian cancer (A2780) cell lines were obtained from ATCC. Cells were maintained in RRMI 1640 medium supplemented with 10 % fetal newborn bovine serum (FBS), 100 units/mL penicillin, and 100 μg/mL streptomycin. Cultures were incubated at 37 °C in a humidified 5 % CO_2_ atmosphere. 

### 3.6. In vitro cytotoxicity assay

WI-38, MRC-5, HCT-8, Bel7402, BGC-823, A549, MCF-7, and A2780 cells were seeded in 96-well microtiter plates at 1,200 cells/well. After 24 h, the tested compounds were added to the systems. After 96 h of drug treatment, cell viability was determined by measuring the metabolic conversion of MTT (3-[4, 5-dimethylthiazol-2-yl]-2, 5-diphenyltetrazolium bromide) into purple formazan crystals by active cells. MTT assay results were read using a MK 3 Wellscan (Labsystem Drogon) plate reader at 570 nm. All compounds were tested in five concentrations and were dissolved in 0.1 % DMSO each well. Each concentration of the compounds was tested in three parallel wells. IC_50_ values were calculated using Microsoft Excel software.

### 3.7. Spectral Data

*Monapurone A*** (1)**: yellow oil; EI-MS *m*/*z* 330 [M]^+^; HREI-MS *m*/*z* 330.1827 [M]^+^ (calc. for C_20_H_26_O_4_, 330.1831); UV (MeOH) λ_max_ (log ε) 352(4.5), 284 (2.2), 230 (4.1) nm; CD (MeOH) λ_max_ (Δε) 258(+0.28), 326 (-6.4) and 369 (+5.5) nm; IR (KBr) ν_max_ 3,403, 1,712, 1,669, 1,618, 1,569, 1,547, 1,450, 1,434, 1,378, 1,219, 1,170 cm^-1^; for ^1^H- and ^13^C-NMR data, see Table 1 and Table 2.

*Monapurone B*** (2):** yellow oil; EI-MS *m*/*z* 344 [M]^+^; HREI-MS *m*/*z* 344.1993 [M]^+^ (calc. for C_21_H_28_O_4_, 344.1988); UV (MeOH) λ_max_ (log ε) 343(4.4), 280 (2.0), 228 (4.0) nm; CD (MeOH) λ_max_ (Δε) 259(+0.22), 334 (-6.6) and 357 (+5.2) nm; IR (KBr) ν_max_ 1,681, 1,661, 1,455, 1,384, 1,277, 1,222, 1,136 cm^-1^; for ^1^H- and ^13^C-NMR data, see Table 1 and Table 2.

*Monapurone C*** (3):** yellow oil; EI-MS *m*/*z* 344 [M]^+^; HREI-MS *m*/*z* 344.1995 [M]^+^ (calc. for C_21_H_28_O_4_, 344.1988); UV (MeOH) λ_max_ (log ε) 345(4.4), 282 (2.1), 229 (4.1) nm; CD (MeOH) λ_max_ (Δε) 265(+0.31), 321(-6.7) and 360 (+5.7) nm; IR (KBr) ν_max_ 1,682, 1,646, 1,609, 1,478, 1,456, 1,411, 1,384, 1,284, 1,162 cm^-1^; for ^1^H- and ^13^C-NMR data, see Table 1 and Table 2.

## 4. Conclusions

As part of our program to systematically assess the chemical constituents of the cytotoxic fraction of *Monascus* *purpureus* metabolites, three new azaphilones, including two stereoisomers, monapurones A–C (**1–3**) were isolated and identified. In the *in* *vitro* cytotoxic assays, monapurones A–C showed potent selective cytotoxicity against the A549 human cancer cell line with IC_50_ value of 3.8, 2.8 and 2.4 μM respectively, while posing no toxicity to normal MRC-5 and WI-38 cells at the same concentration. 

## Supplementary Materials

Supplementary information can be seen at http://www.mdpi.com/1420-3049/15/3/1958/s1/.
