# Supplementary material for: New Cytotoxic Azaphilones from Monascus purpureus-Fermented Rice (Red Yeast Rice)
Source: Molecules. 2010 Mar 18;15(3):1958–66. doi: 10.3390/molecules15031958 (PMC6257306; doi:10.3390/molecules15031958)

Compound 1 H-NMR IN CD_3Cl_3

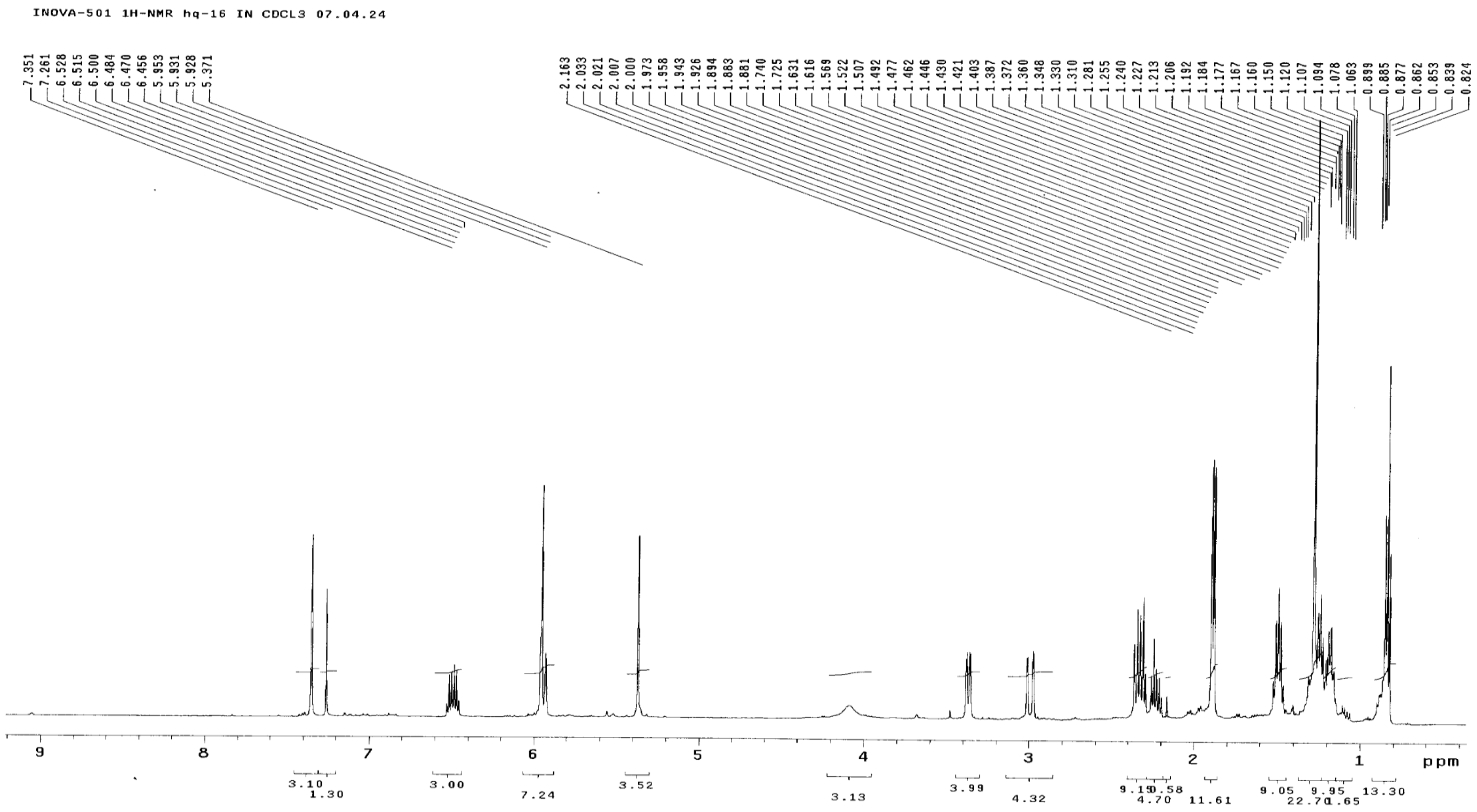

Compound 1 H-NMR IN CD_3COCD_3

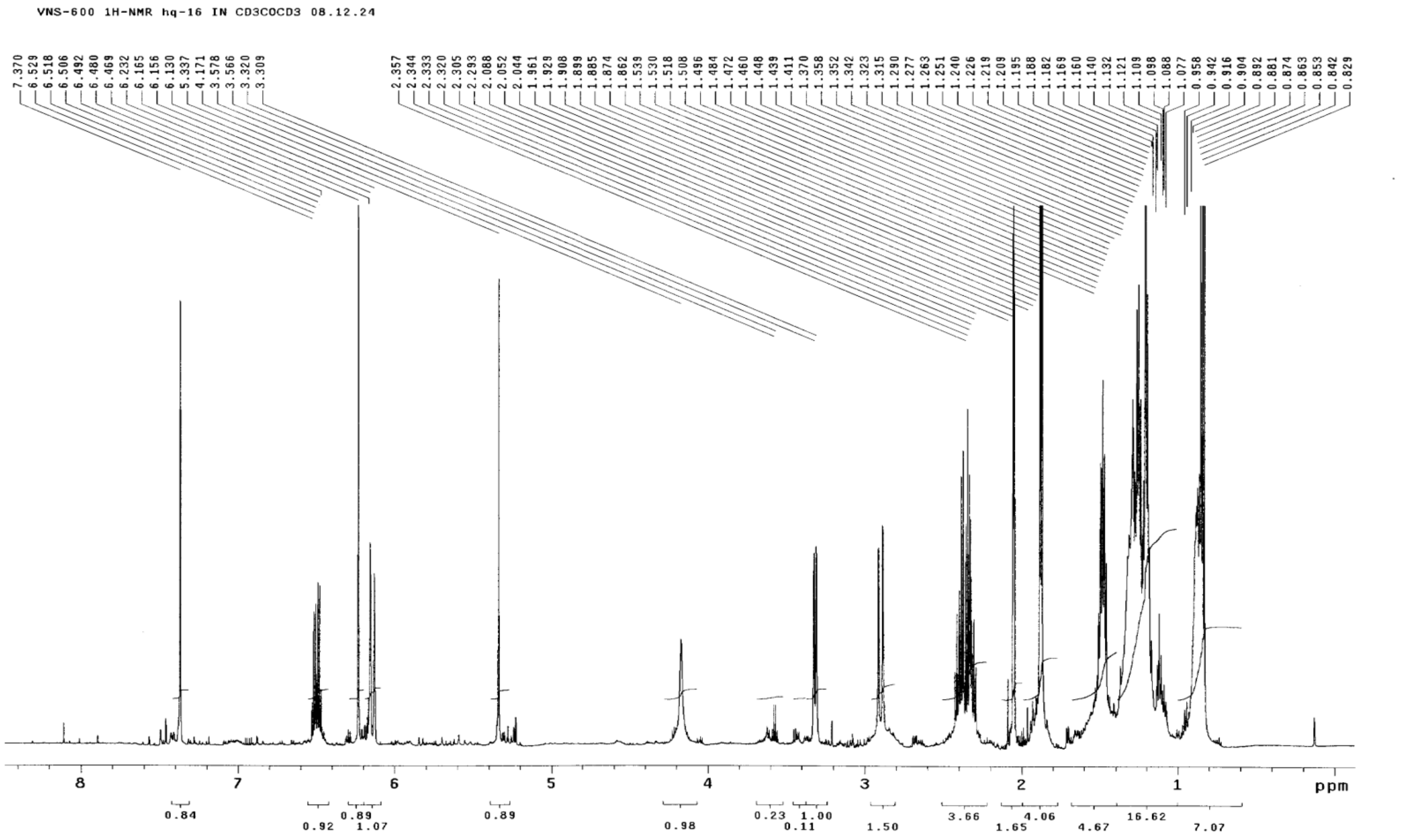

Compound 1 C-NMR IN CD_3Cl_3

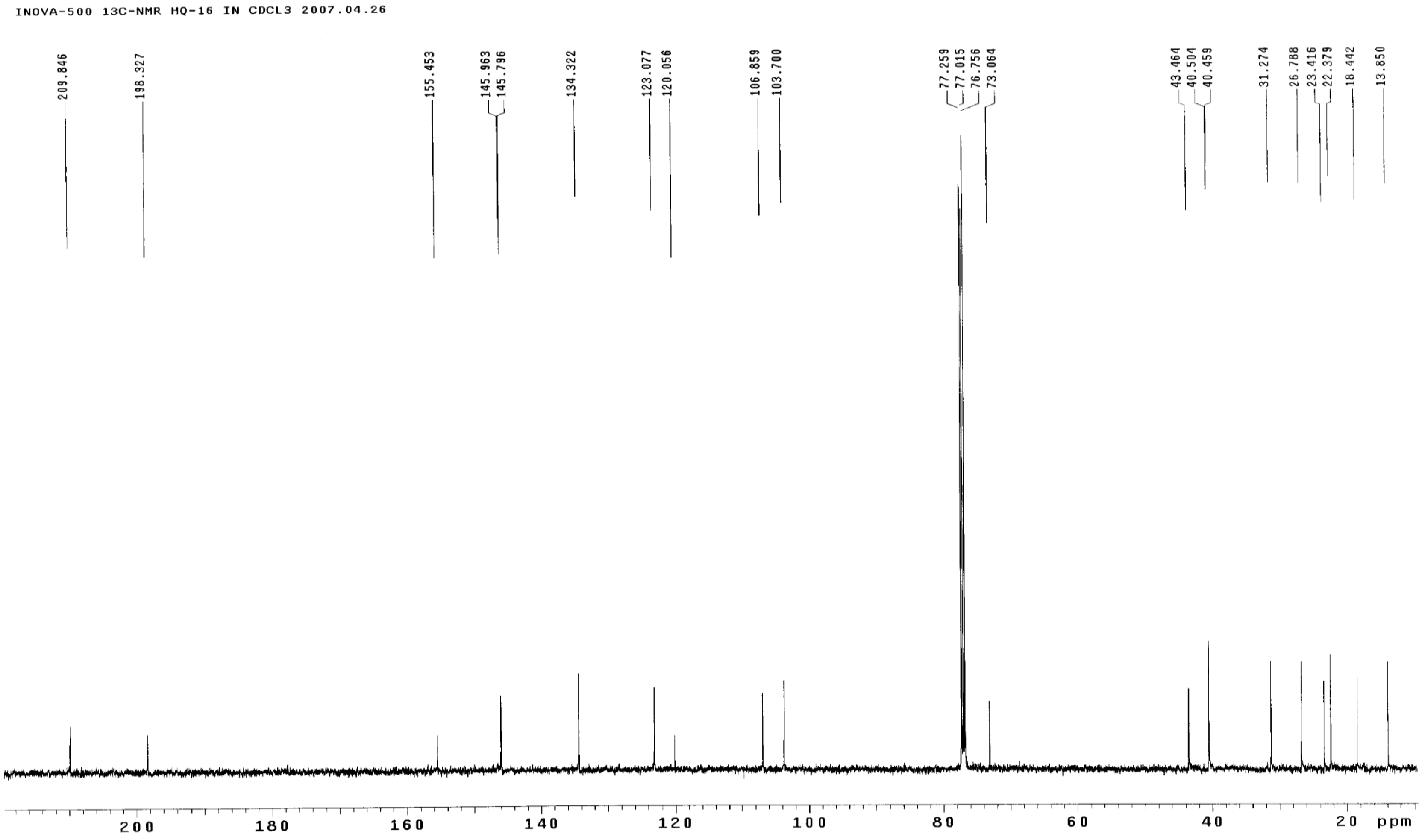

Compound 1 DEPT-NMR IN CD_3Cl_3

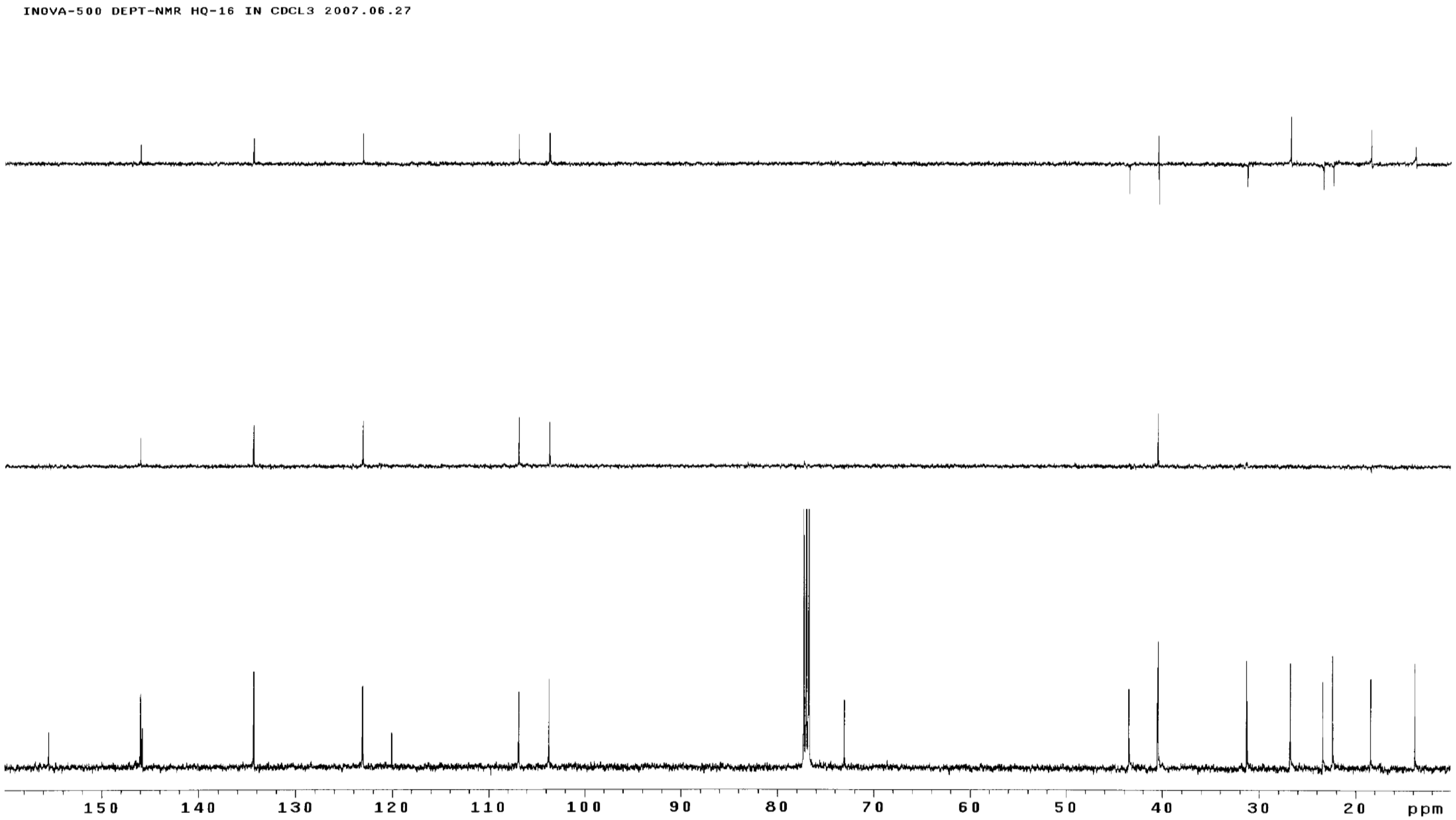

Compound 1 gCOSY IN CD_3Cl_3

INOVA-501 gCOSY HQ-16 IN CDCL3 07.05.21

Solvent: CDCl3
Temp. 25.0 C / 298.1 K
INOVA-500 "IMM-501"

Relax. delay 1.000 sec
Acq. time 0.126 sec
Width 4070.6 Hz
2D Width 4070.6 Hz
2 repetitions
256 increments
OBSERVE H1, 499.7702080 MHz
DATA PROCESSING
Sine bell 0.063 sec
F1 DATA PROCESSING
Sine bell 0.031 sec
FT size 1024 x 1024
Total time 10 min, 17 sec

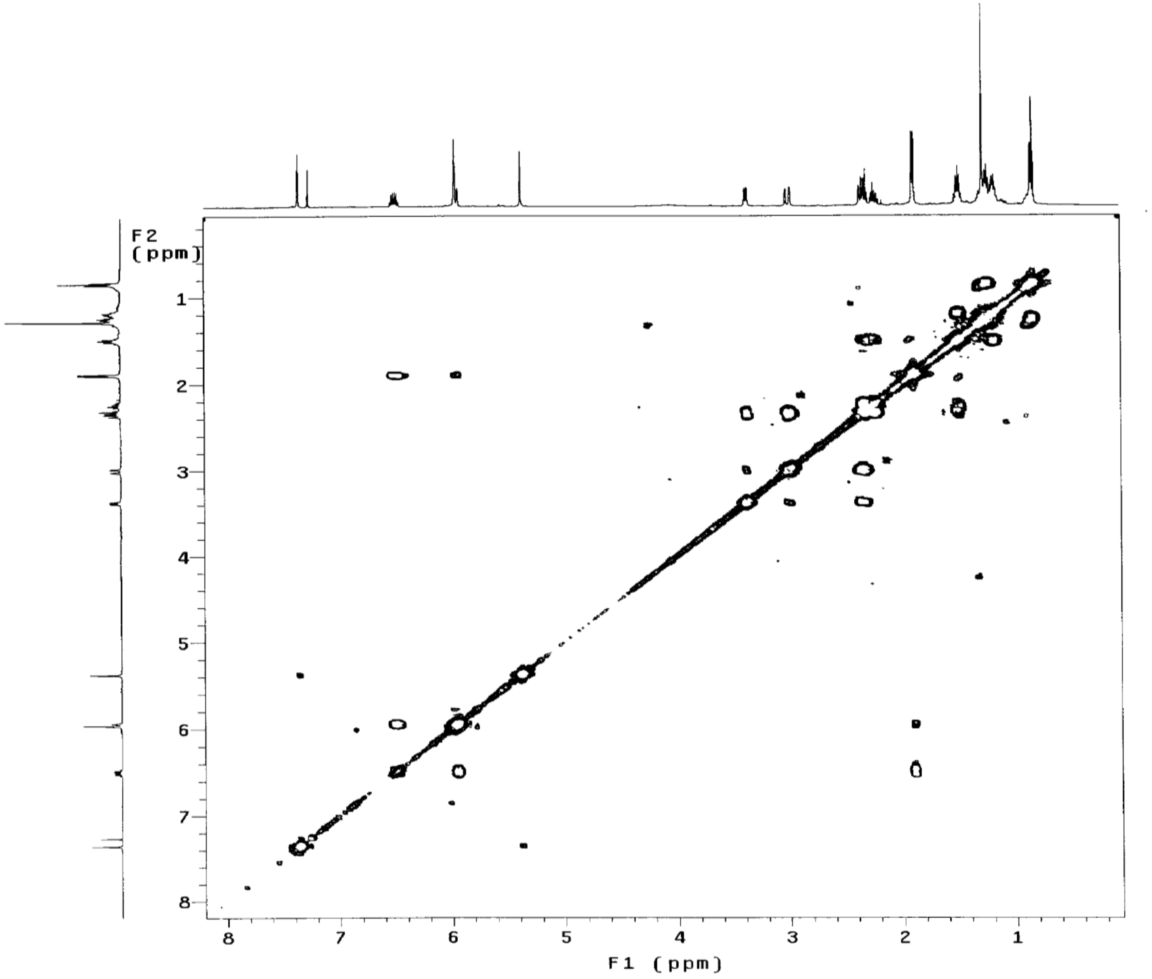

Compound 1 gCOSY IN CD_3COCD

VNS-600 gCOSY hq-16 IN CD3COCD3 08.12.24

```
 Solvent: acetone
 Ambient temperature
Operator: vnmr2
VNMRS-600  "wormhole"

 Relax. delay 1.301 sec
 Acq. time 0.205 sec
 Width     10000.0 Hz
 2D Width 10000.0 Hz
 2 repetitions
 256 increments
OBSERVE   H1, 599.6981281 MHz
DATA PROCESSING
 Sine bell 0.099 sec
F1 DATA PROCESSING
 Sine bell 0.009 sec
FT size 4096 x 4096
Total time 13 min, 28 sec
```

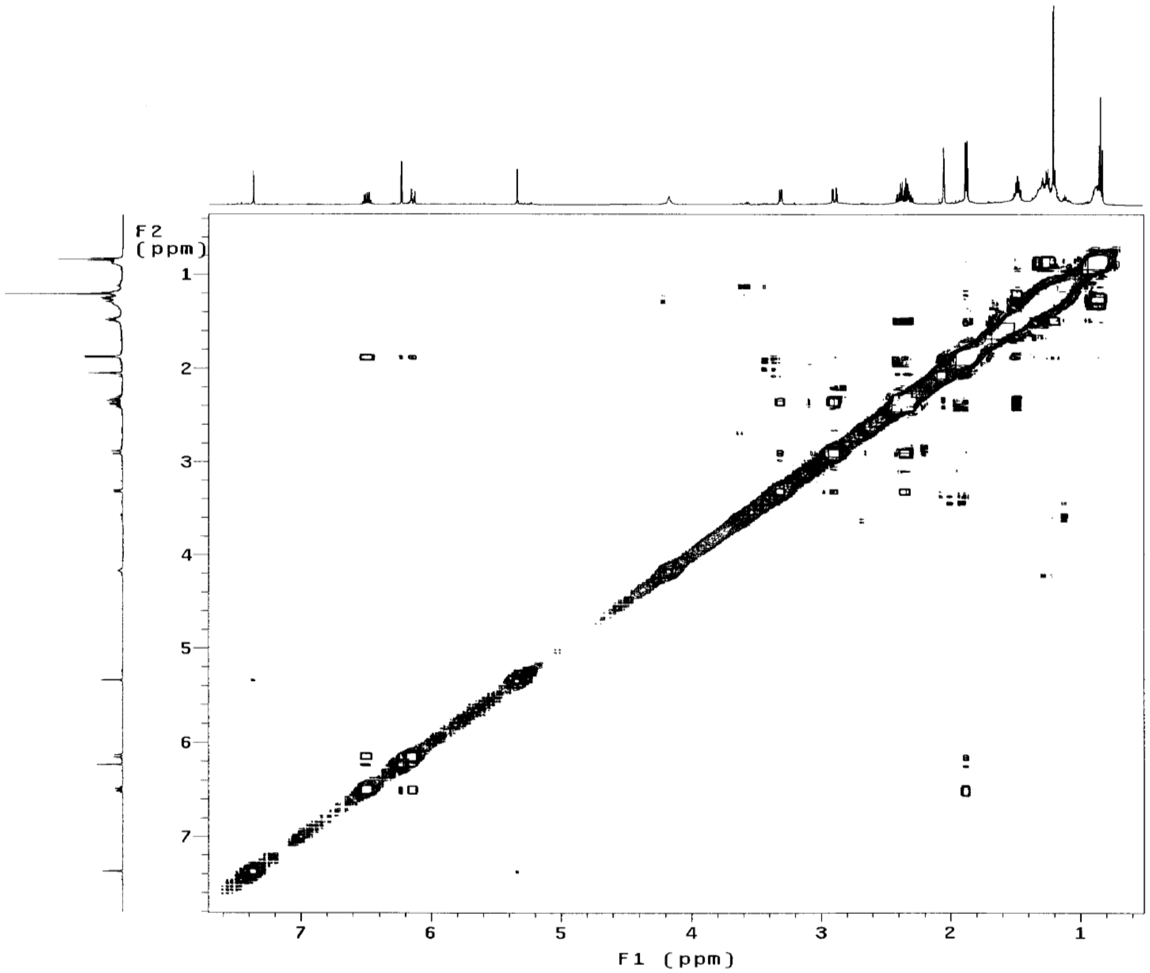

Compound 1 gHMQC IN CD_3Cl_3

INOVA-501 1H-NMR HQ-16 IN CDCL3 07.05.21

Solvent: CDCl3
Temp. 25.0 C / 298.1 K
User: 1-14-87
INOVA-500 "IMM-501"

Relax. delay 1.000 sec
Acq. time 0.213 sec
Width 4797.6 Hz
2D Width 21175.2 Hz
16 repetitions
160 increments
OBSERVE H1, 499.7702080 MHz
DECOUPLE C13, 125.6780857 MHz
Power 48 dB
on during acquisition
off during delay
GARP-1 modulated
DATA PROCESSING
Sine bell 0.042 sec
F1 DATA PROCESSING
Sine bell 0.004 sec
FT size 2048 x 4096
Total time 55 min, 32 sec

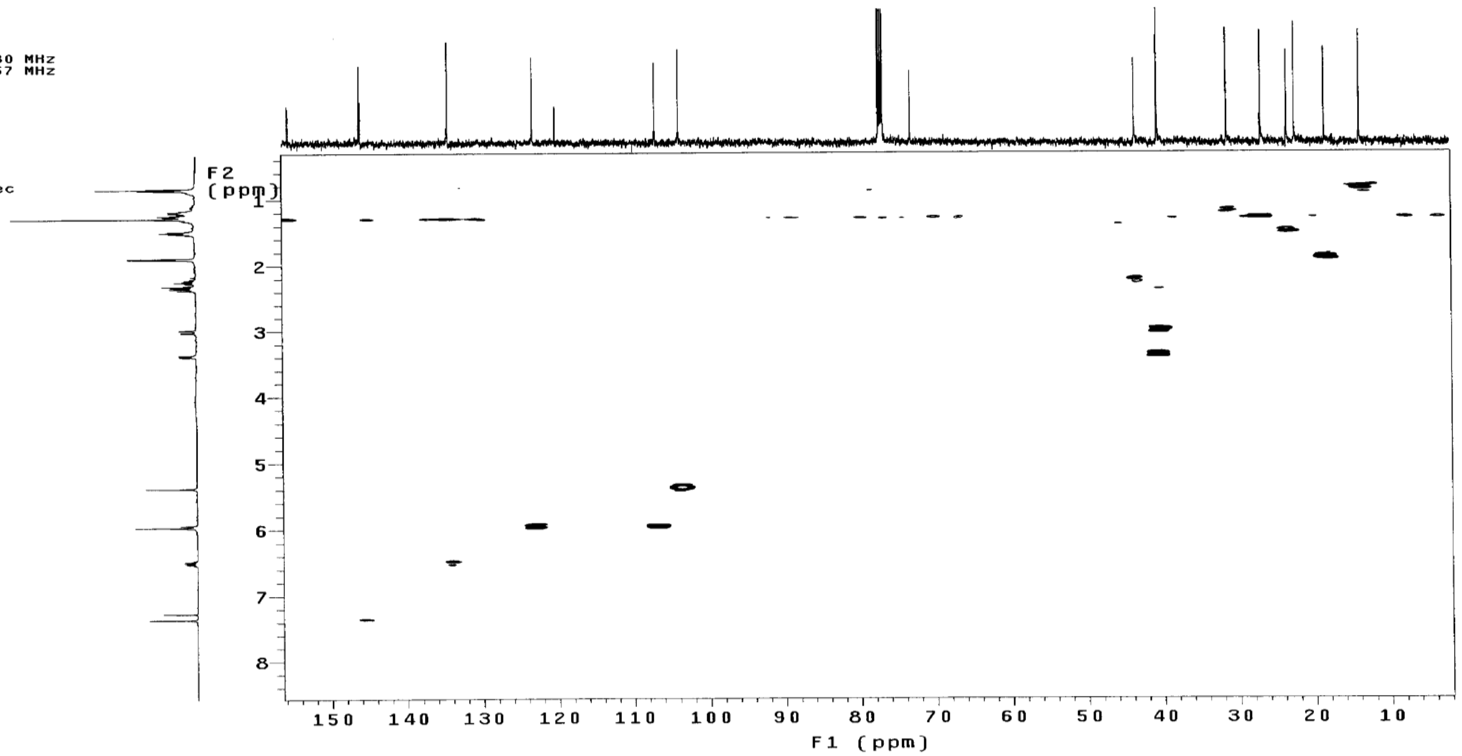

Compound 1 gHMBC IN CD_3Cl_3

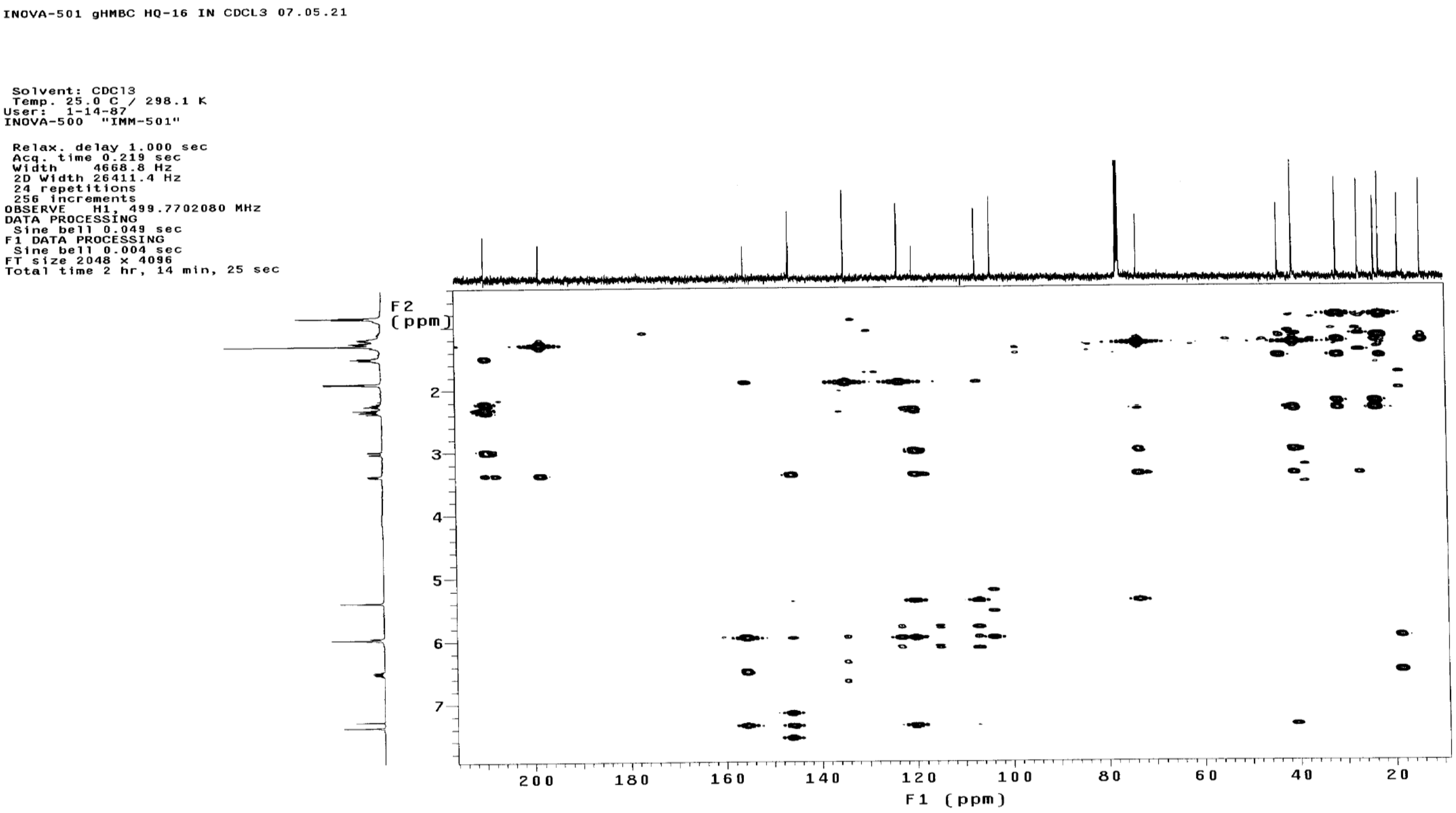

Compound 1 NOESY IN CD_3Cl_3

INOVA-501 NOESY HQ-16 IN CDCL3 07.07.04

Solvent: CDCl3
Temp. 25.0 C / 298.1 K
INOVA-500 "IMM-501"

Relax. delay 1.600 sec
Mixing 0.800 sec
Acq. time 0.132 sec
Width 3887.5 Hz
2D Width 3887.5 Hz
8 repetitions
2 x 200 increments
OBSERVE H1, 499.7702083 MHz
DATA PROCESSING
Gauss apodization 0.031 sec
F1 DATA PROCESSING
Gauss apodization 0.008 sec
FT size 2048 x 2048
Total time 2 hr, 18 min, 28 sec

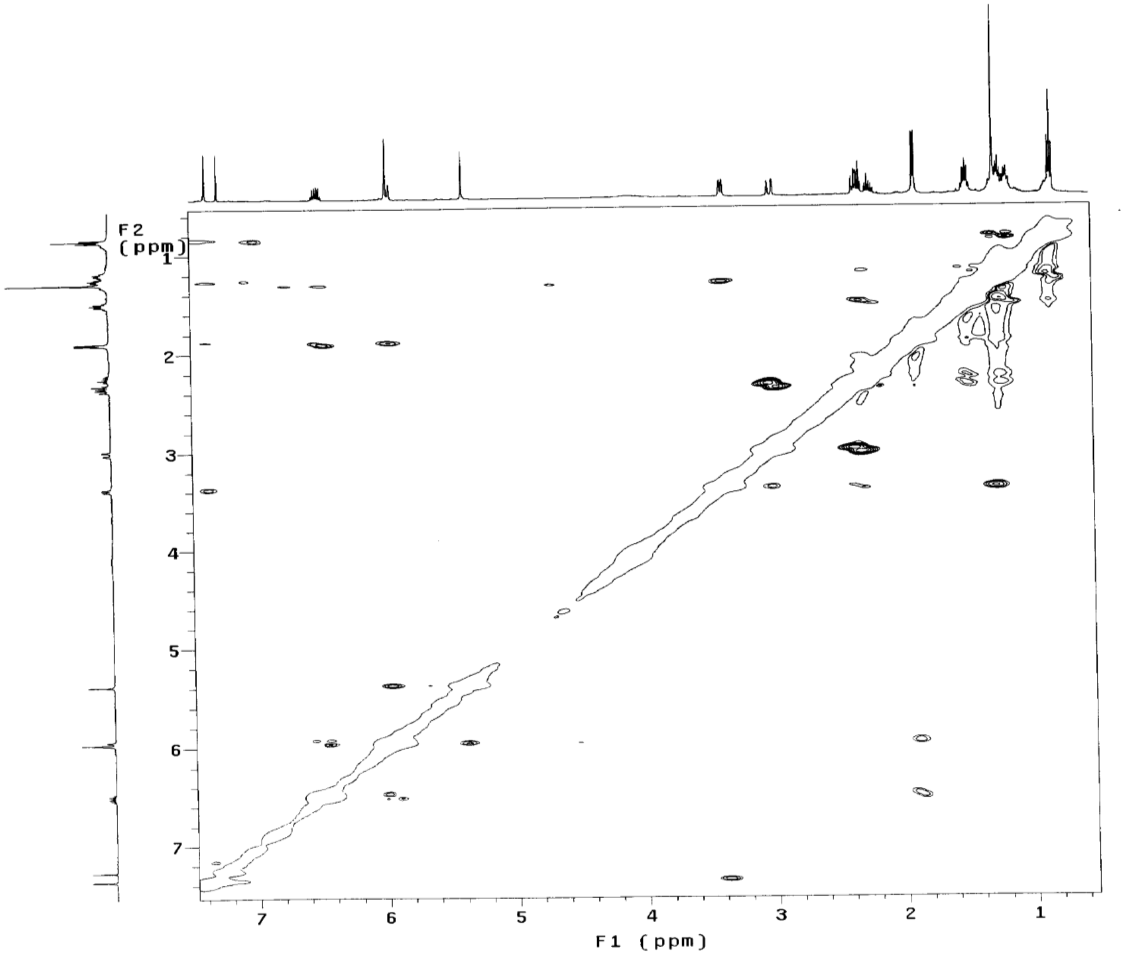

Compound 1 NOESY IN CD_3COCD_3

VNS-600 NOESY hq-16 IN CD3COCD3 08.12.24

Solvent: acetone
Ambient temperature
Operator: vnmr2
VNMRS-600 "wormhole"

Relax. delay 1.200 sec
Mixing 0.600 sec
Acq. time 0.197 sec
Width 5186.7 Hz
2D Width 5186.7 Hz
16 repetitions
2 x 200 increments
OBSERVE H1, 599.6981281 MHz
DATA PROCESSING
Gauss apodization 0.034 sec
F1 DATA PROCESSING
Gauss apodization 0.010 sec
FT size 4096 x 4096
Total time 3 hr, 38 min, 3 sec

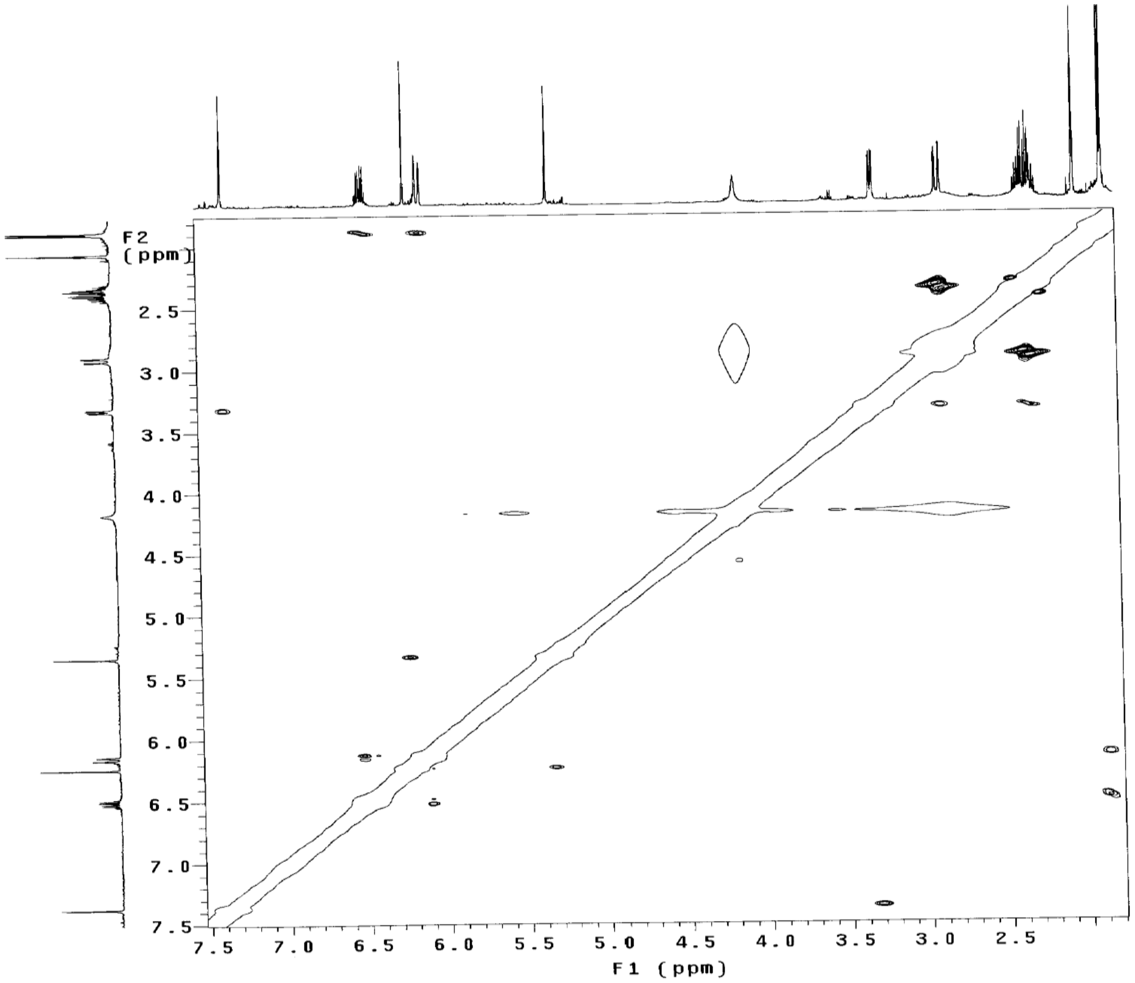

Compound 1 CD

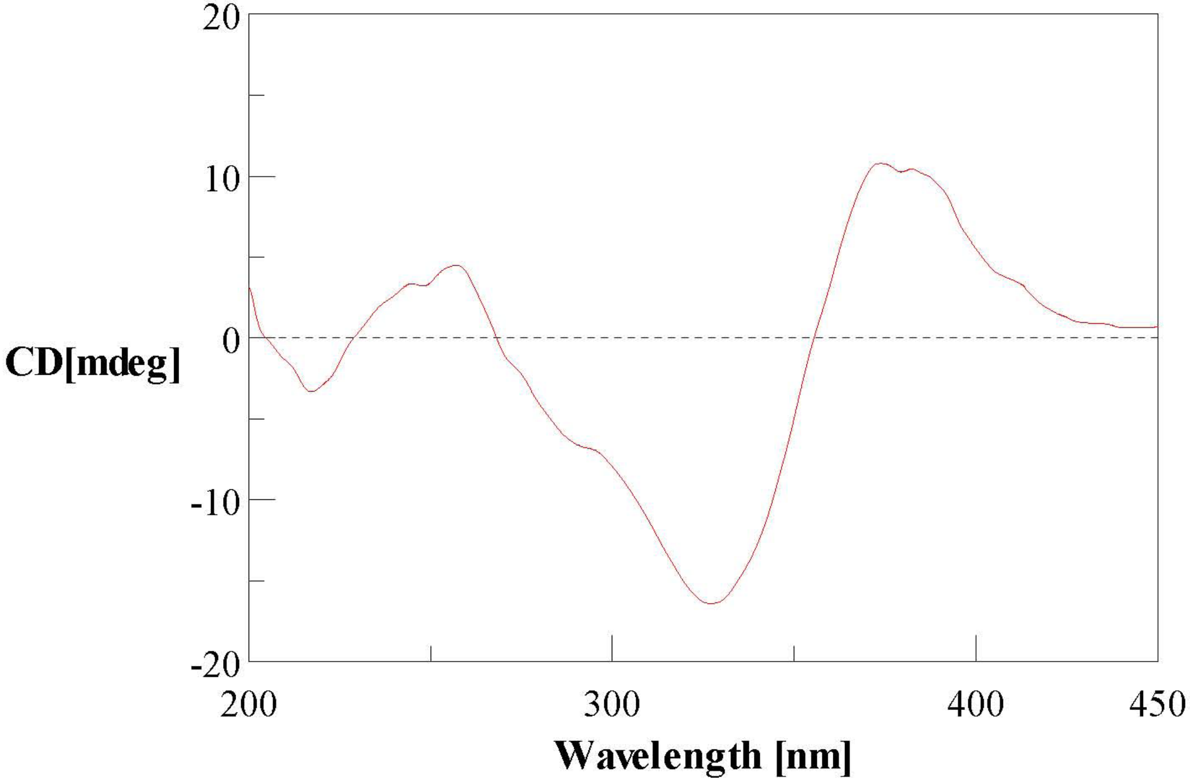

Compound 2 H-NMR IN CD_3Cl_3

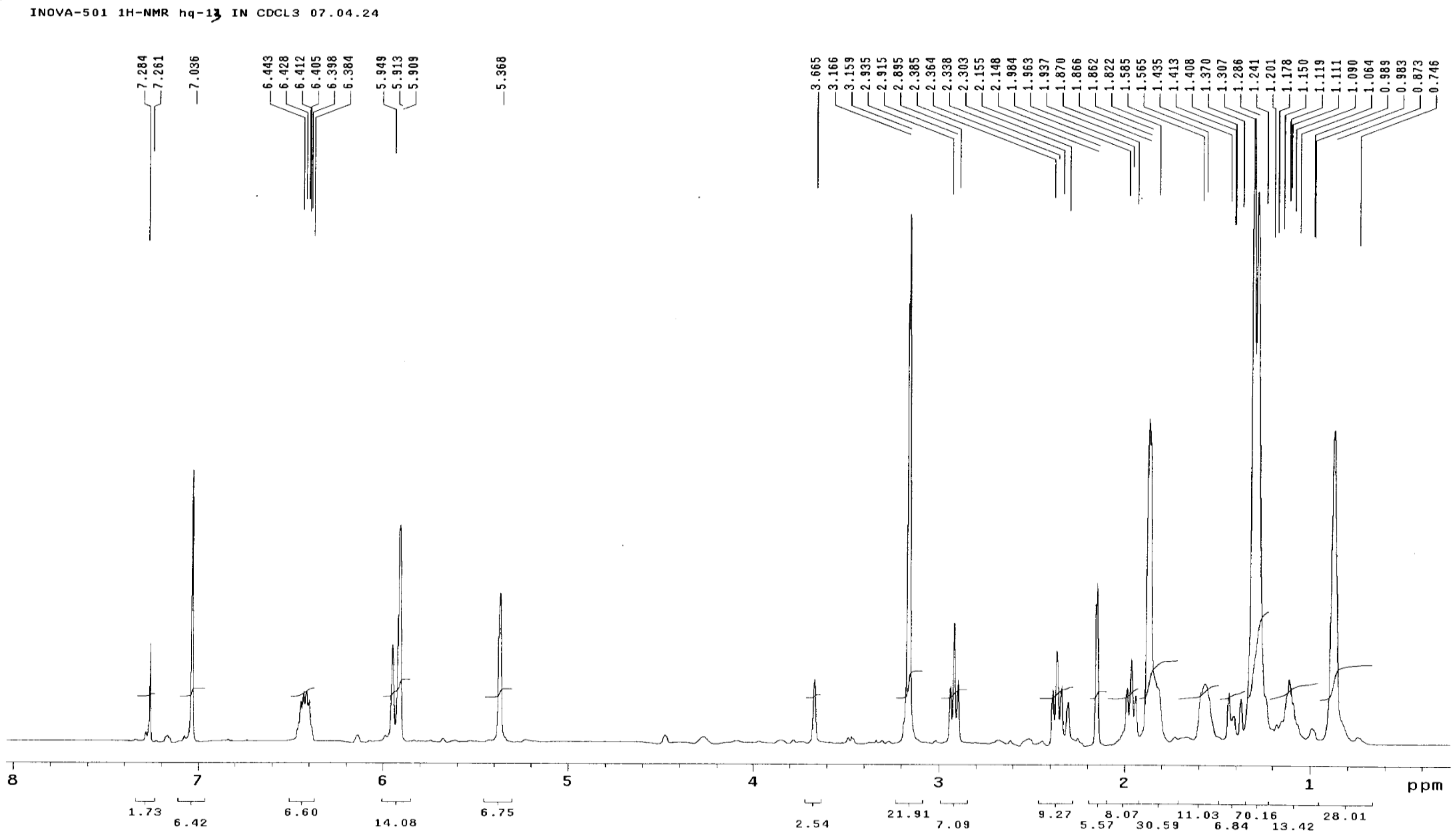

Compound 2 C-NMR IN CD_3Cl_3

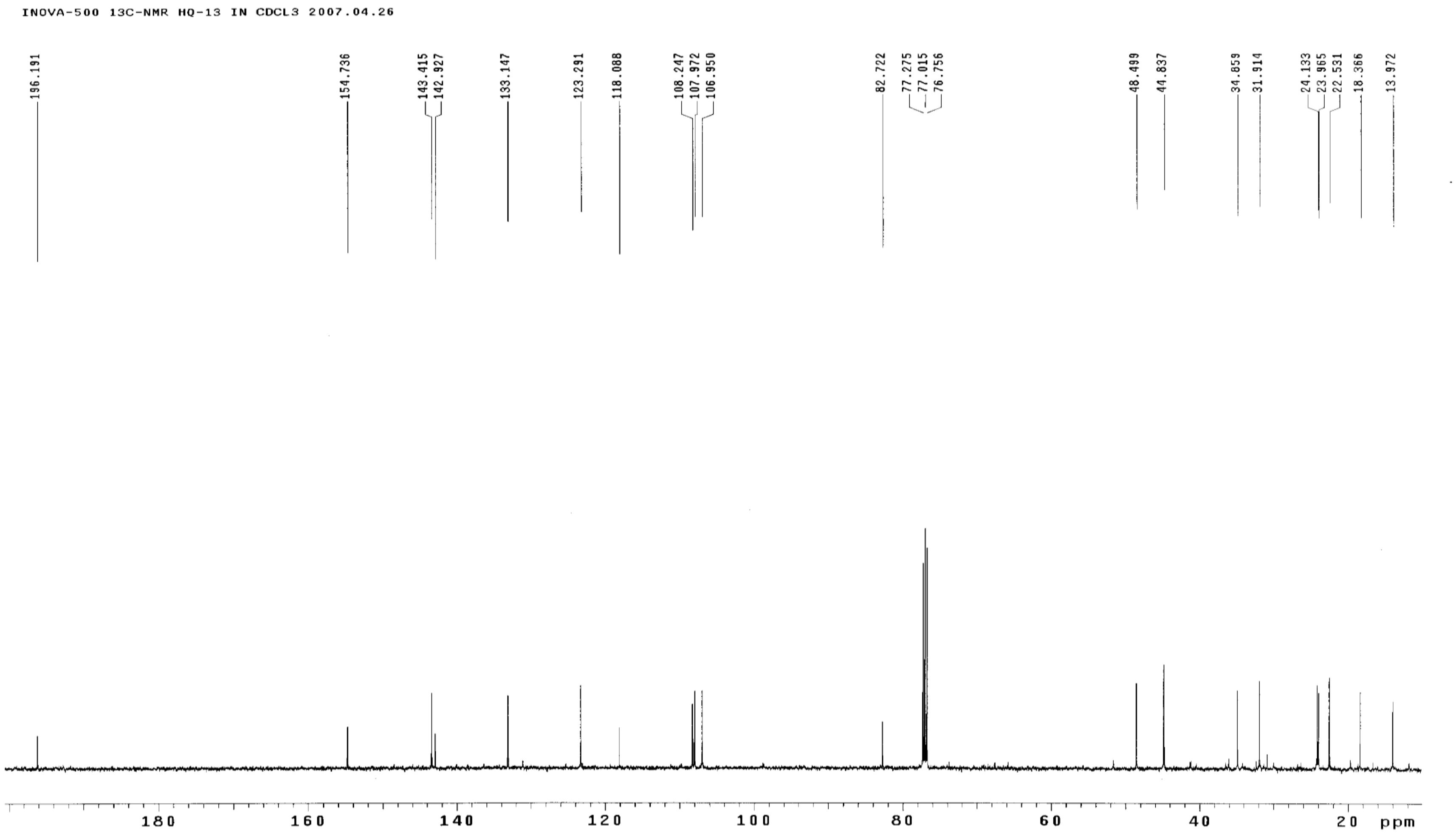

Compound 2 gHMBC IN CD_3Cl_3

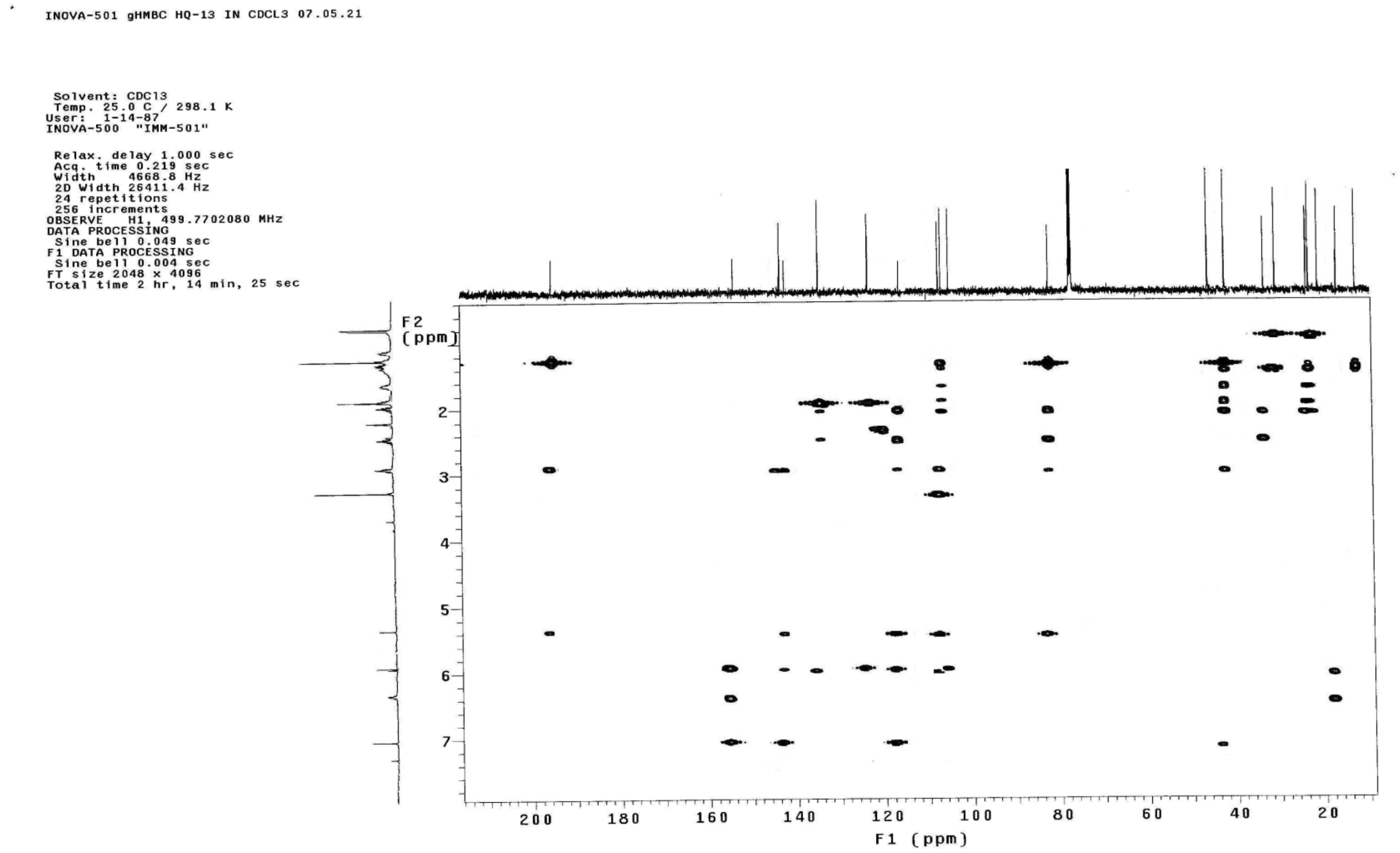

Compound 2 NOESY IN CD_3Cl_3

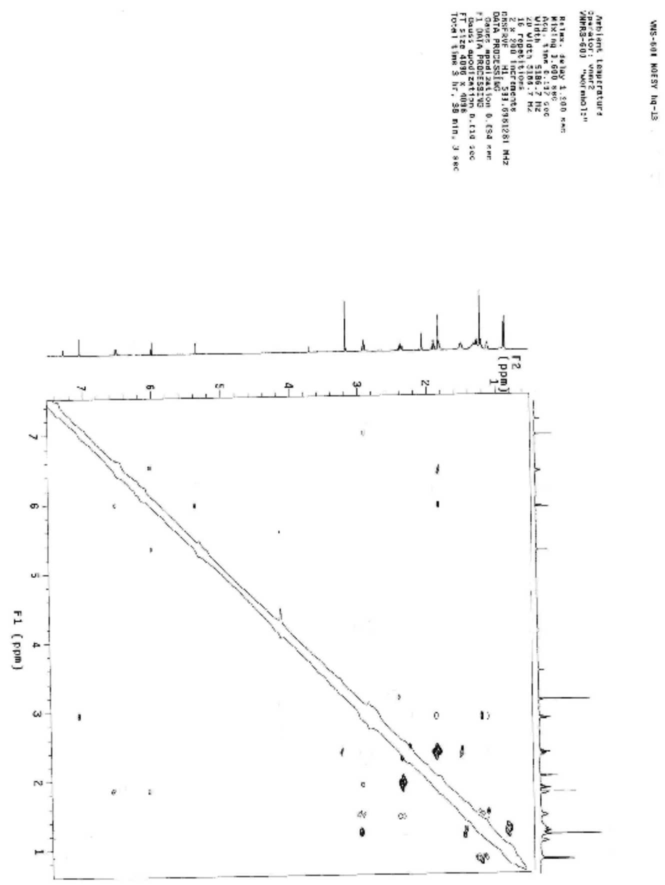

Compound 2 CD

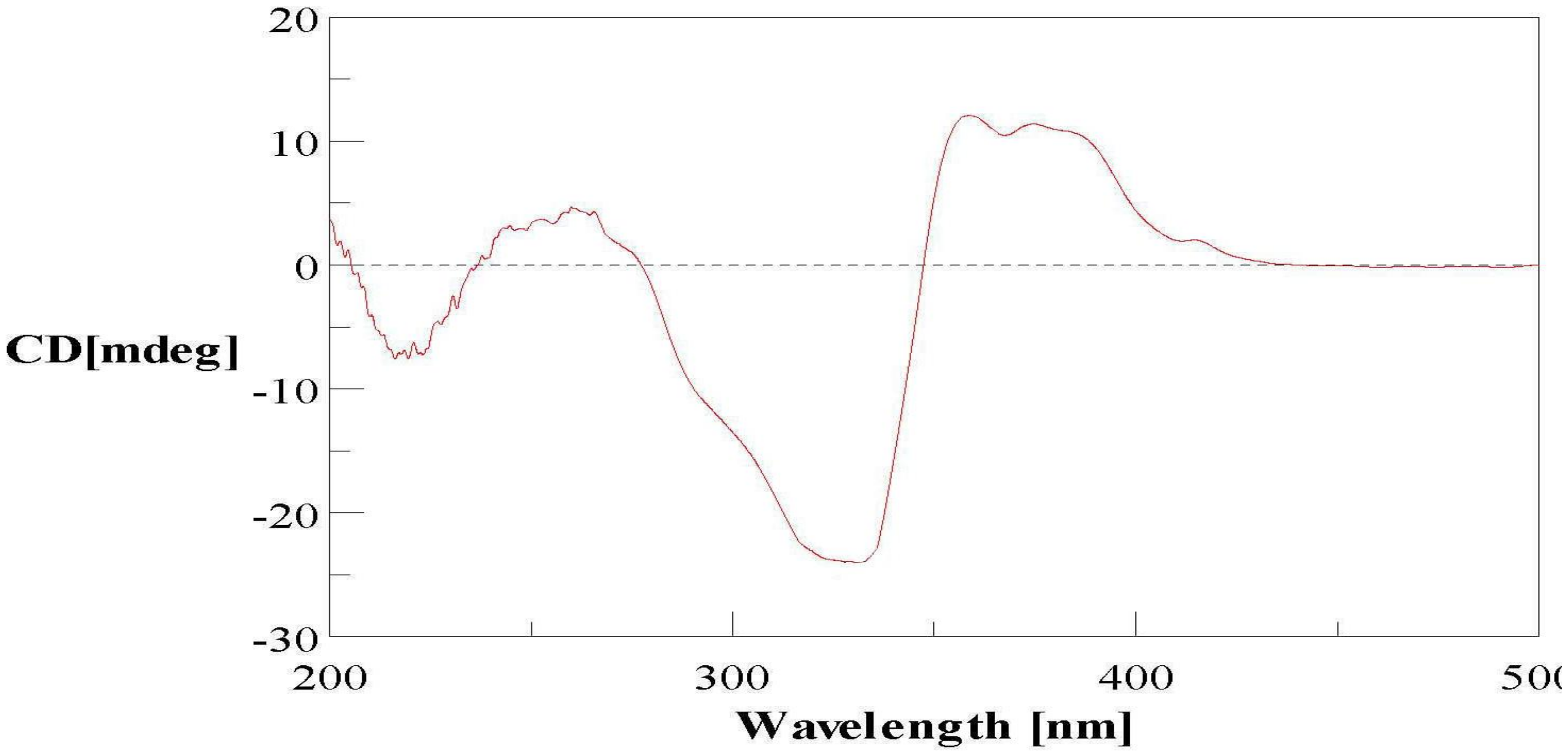

Compound 3 H-NMR IN CD_3Cl_3

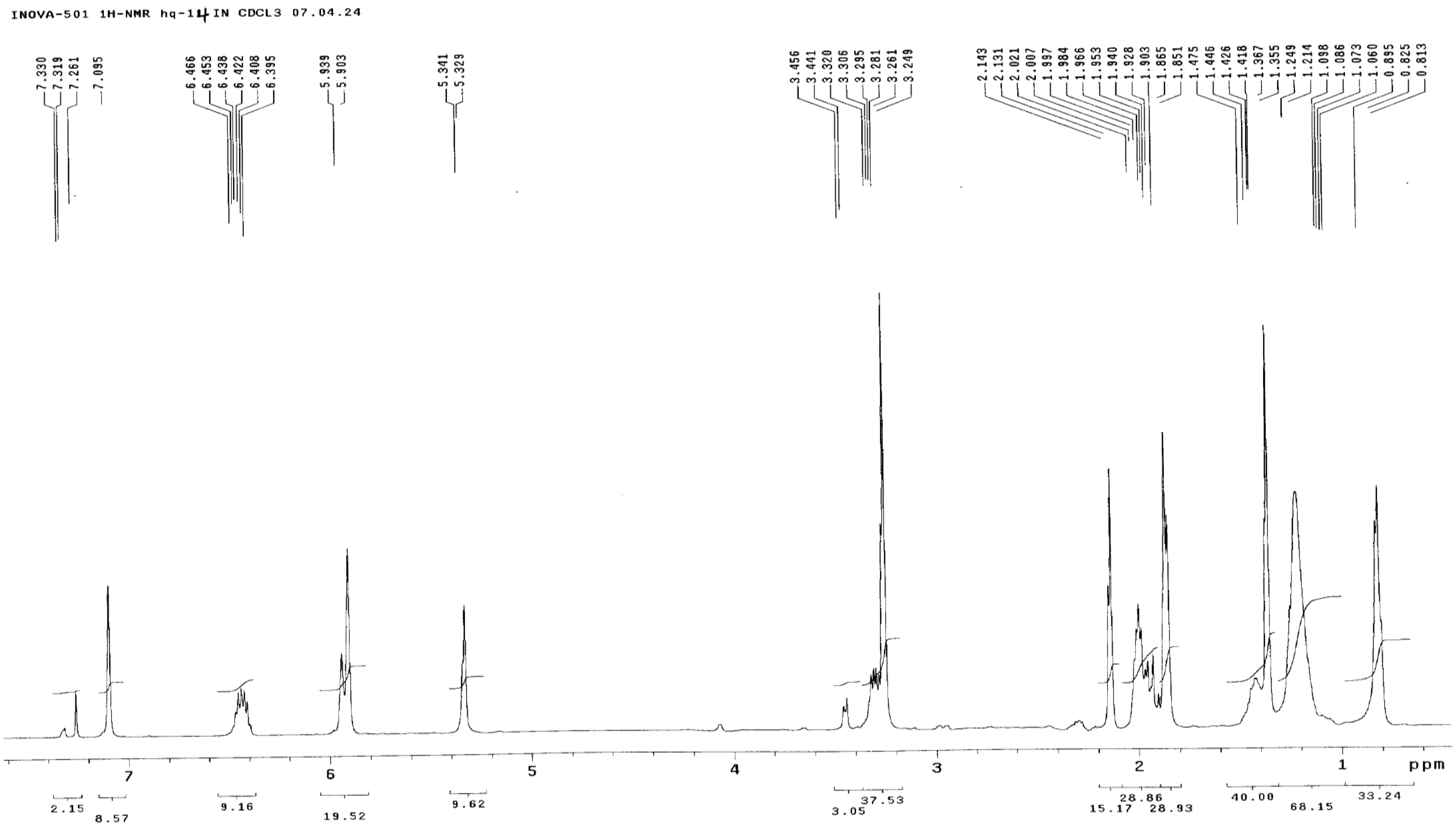

Compound 3 C-NMR IN CD_3Cl_3

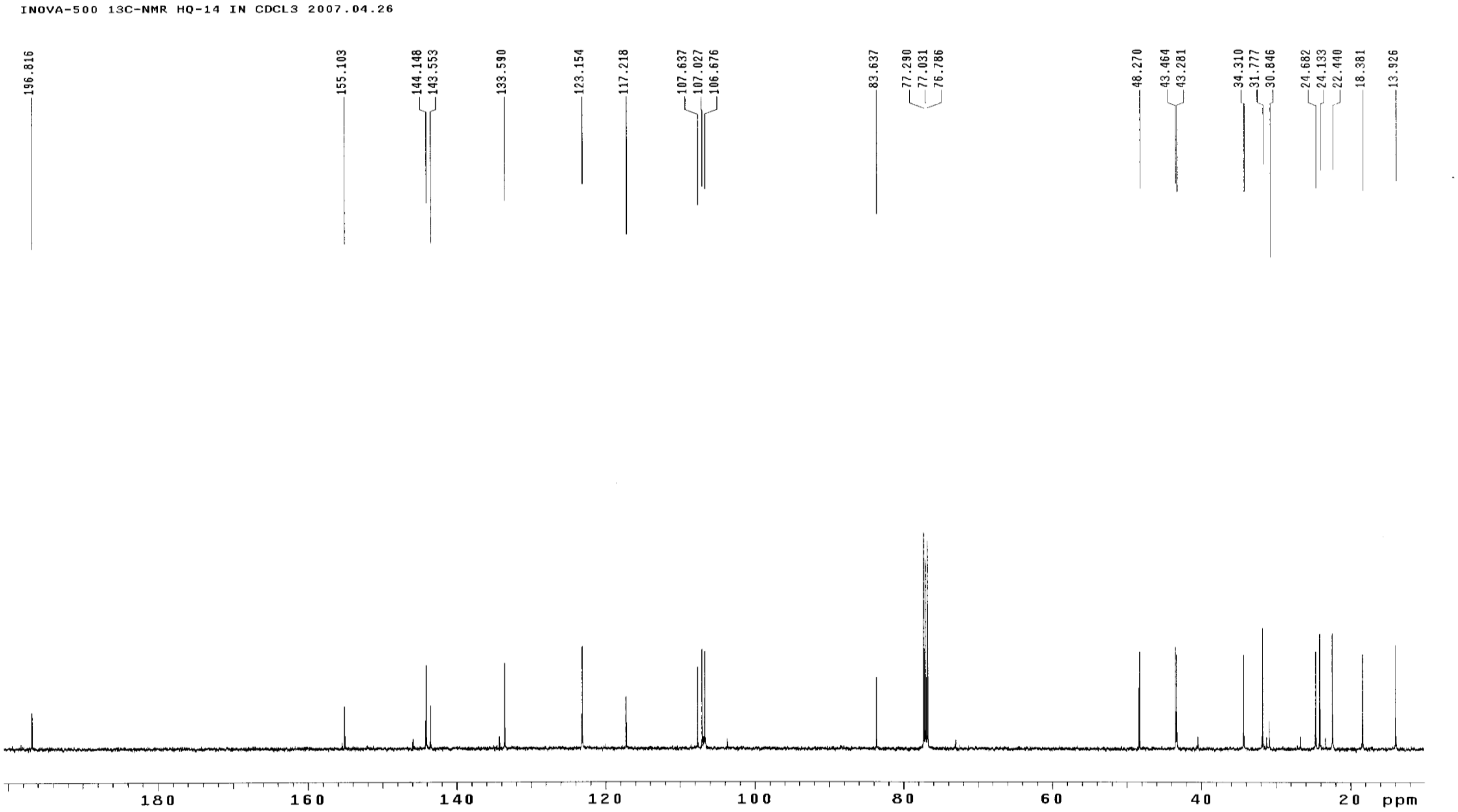

Compound 3 gHMBC IN CD_3Cl_3

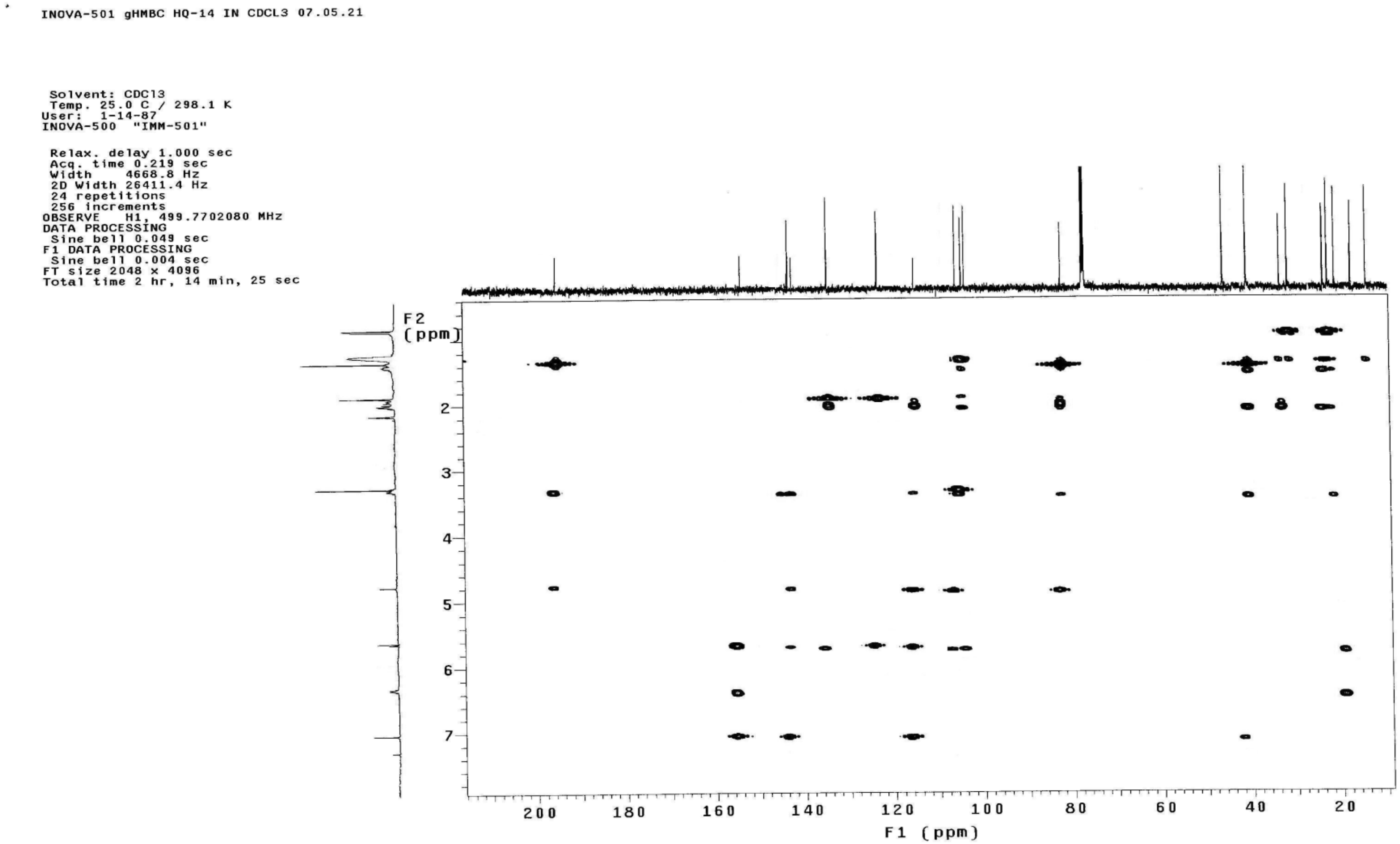

Compound 3 NOESY IN CD_3Cl_3

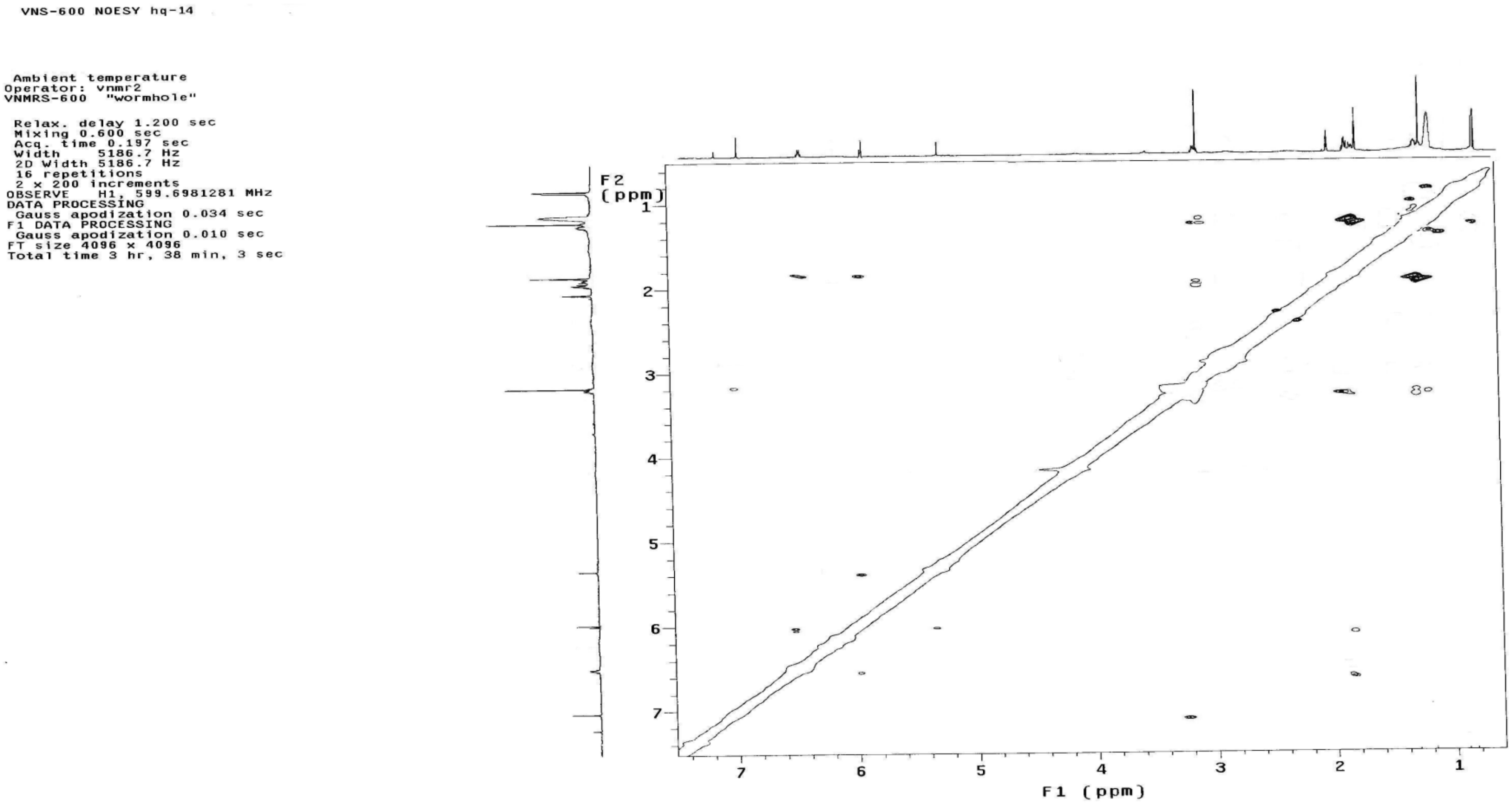

Compound 3 CD IN CD_3Cl_3

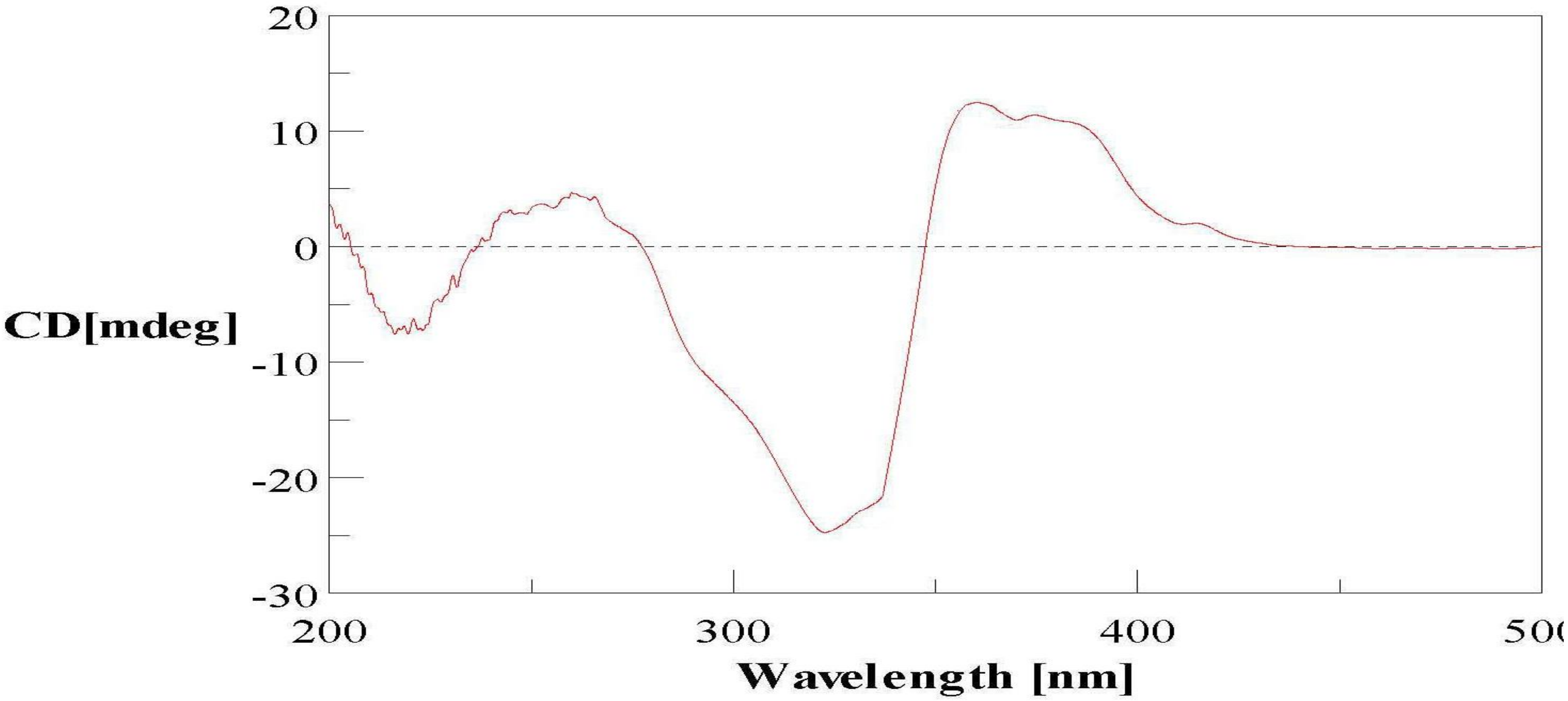

Supplement: Supplementary file 1 [file molecules-15-01958-s001.pdf]
